# Design and Experiment of Satellite-Terrestrial Integrated Gateway with Dynamic Traffic Steering Capabilities for Maritime Communication

**DOI:** 10.3390/s23031201

**Published:** 2023-01-20

**Authors:** Hyounhee Koo, Jungho Chae, Wooseong Kim

**Affiliations:** 1 Department of Computer Engineering, Graduate School of Gachon University, 1342 Seongnamdaero, Sujeong-gu, Seongnam 13120, Republic of Korea; 2 SyncTechno Inc., Rm 914/918, 311 Gangnamdaero, Seocho-gu, Seoul 06628, Republic of Korea

**Keywords:** multi-rat, gateway, autonomous surface ship, maritime, MRGW, 5G-Advanced, 6G

## Abstract

This study presents the architectural design and implementation of a multi-RAT gateway (MRGW) supporting dual satellite and terrestrial connectivity that enables moving maritime vessels, such as autonomous surface ships, to be connected to multiple radio access networks in the maritime communication environment. We developed an MRGW combining LTE and very-small-aperture terminal (VSAT) access networks to realize access traffic steering, switching, and splitting functionalities between them. In addition, we developed communication interfaces between the MRGW and end-devices connecting to their corresponding radio access networks, as well as between the MRGW and the digital bridge system of an autonomous surface ship, enabling the MRGW to collect wireless channel information from each RAT end-device and provide the collected data to the digital bridge system to determine the optimal navigation route for the autonomous surface ship. Experiments on the MRGW with LTE and VSAT end-devices are conducted at sea near Ulsan city and the Kumsan satellite service center in Korea. Through validation experiments on a real maritime communication testbed, we demonstrate the feasibility of future maritime communication technologies capable of providing the minimum performance necessary for autonomous surface ships or digitized aids to navigation (A to N) systems.

## 1. Introduction

Maritime communications using maritime frequency bands within the very high frequency (VHF) spectrum became the most widely-used communication system for vessels after Marconi’s success in transatlantic communications with a transmission distance of over 3000 km in 1901 [1]. Currently, commercial maritime communication uses cellular communication based on LTE or 5G technology for vessels along the coast, where vessels are within the coverage of the cellular network. This is also applicable to onboard communication on luxury cruise ships or state-of-the-art ships such as small-size electric ships or autonomous surface ships [2].

Traditional maritime communication technologies may coexist with state-of-the-art technologies under two conditions: the long life expectancy of ships, which is usually >20 or 30 years, and the difference in countrywide communication infrastructures, which requires ships to be equipped with multiple communication interfaces for access networks permitted by a country whose ships visit during their navigation [3,4,5]. In addition, the international maritime organization (IMO) has recently completed a regulatory scoping exercise on Maritime Autonomous Surface Ships (MASS) [6]. The International Association of Marine Aids to Navigation and Lighthouse Authorities (IALA) is developing guidelines for the future development of maritime radio communication, including IMT-2020, which is applicable to their digital transformation, such as autonomous surface ships [7,8]. Since the maritime sector was included in the standardization scope of 3rd Generation Partnership Project (3GPP) in December 2018, use cases and requirements related to the maritime sector have been considered in addition to other use cases and requirements from several vertical industries [9,10,11,12].

In such a maritime communication environment with heterogeneous communication technologies, the support of multiple radio access technologies (multi-RATs) is essential from the perspective of the upcoming digital transformation era in the maritime sector [13,14,15,16,17,18]. There are research works on the support of multi-RAT including LTE and Wi-Fi in terrestrial environments (e.g., in ultra-dense urban area, in smart cities, in vehicular or air-to-ground communications), but their outcomes were mainly based on the simulations and did not provide results based on the real experimental validation with the implemented equipment [19,20,21,22,23].

In this study, we design and implement a multi-RAT gateway (MRGW) with the capabilities of steering, switching, and splitting the access traffic, and provide experimental validation of the proposed MRGW prototype connected to LTE/VAT end-devices in the testbed of the Korean autonomous surface ship (KASS) at sea, near Ulsan city and the Kumsan satellite service center, Republic of Korea.

The key contributions of this study are summarized as follows:

- This study contributes to the literature by proposing the design of an overall system model applicable for the maritime communication environment where an autonomous surface ship can exchange information with an on-land control center through an MRGW that enables intelligent routing among multi-RATs.
- Another contribution is the proposition of a detailed design of an MRGW with several key features adopted from open-source software and newly developed to monitor the radio channel status of each RAT end-device.
- Finally, this study demonstrates that the MRGW can enable the interworking between two heterogeneous RAT end-devices and support real-time video streaming through experiments on a real maritime testbed in three test scenarios.

This paper is organized as follows. Section 2 describe the related works and Section 3 introduces the overall multi-RAT supporting communication system model applicable to the maritime communication environment where autonomous surface ships exchange information with an on-land control center and neighboring ships or digital transform aids to navigation equipment using multi-RATs. Section 4 describes the design of the MRGW with an interface for each RAT end-device. Section 5 describes the system configurations for the field test environment and three test deployment scenarios, each of which comprises four or five steps for the experiment execution process. Section 6 analyzes the experimental outcome on the basis of test results. Finally, the conclusions and future research directions are discussed in Section 6.

## 2. Related Works

For the modernization of maritime communications, the transition to digital technologies has begun in the maritime sector and the existing systems are being enhanced and new technologies continue to emerge [4]. The VHF data exchange system (VDES) and Navigational Data (NAVDAT) were developed as maritime communication systems supporting the e-navigation concept and the modernization of the Global Maritime Distress and Safety System (GMDSS) [24,25,26].

The maritime autonomous surface ship (MASS) is also one of the main research streams for the digital transformation of the maritime sector [27,28,29] and there are ongoing works to enable advanced communication technologies including satellites, Unmanned Aerial Vehicles (UAVs), and IMT systems (e.g., 5G, 6G) with improved coverage and data rates to be applied for the MASS [30,31,32,33,34].

Satellite communication has been widely employed in the maritime sector because of the communication coverage and the accessible network infrastructure in the ocean, and traditionally provides limited applications due to the long propagation delay and affordable data rate. However, the recent technological advancement is ongoing for the integration of satellites into the 5G or 6G networks [35,36,37,38].

The support of the multi-RATs is one of the key requirements of several vertical industries, including the maritime sector, because of the heterogeneous networks. The multi-connectivity combining different access technologies (e.g., cellular, satellite, Wi-Fi) continue to be enhanced for seamless connection in diverse service scenarios over 5G or 6G systems [39,40,41,42].

## 3. System Model

**Figure 1:**
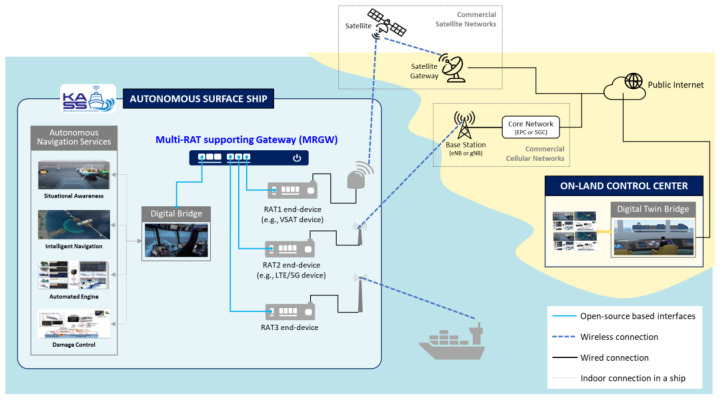
System Model.

Our system model for the maritime communication environment is depicted in Figure 1. It enables an autonomous surface ship to exchange the information with an on-land control center via external communication systems accessible from a ship near the shore or at sea.

A commercial satellite access network (e.g., very-small-aperture terminal (VSAT) system) and a cellular access network (e.g., LTE system) are assumed to be accessible by each subscribed RAT end-device connected to the MRGW. In addition, the digital twin bridge of the on-land control center communicates with the digital bridge of the autonomous surface ship through the commercial satellite or cellular access network. This communication channel can be established through public Internet or a closed network (e.g., 5G private network) if available commercially or proprietarily deployed near a shore or at port.

The MRGW handles the acquisition of wireless channel information from each RAT end-device and the periodic provision of that information to the digital bridge. It provides the digital bridge of the autonomous surface ship with information about the best RAT at the current location that an autonomous surface ship is navigating. The MRGW provides two types of interfaces:

- An interface between the digital bridge and the MRGW to provide the wireless channel status measurement of each RAT end-device to the digital bridge, and to enable the MRGW to obtain the navigation information of the autonomous ship managed by the digital bridge;
- An interface between the MRGW and each RAT end-device, which depends on the communication protocol supported by each RAT end-device.

As 5G-Advanced or 6G communication systems are expected to provide seamless connection anywhere (e.g., in either the maritime or terrestrial sector), with the support of multi-RATs, the current commercial communication networks will evolve accordingly [43,44,45]. Therefore, the MRGW is designed to be capable of providing interfaces to future RAT end-devices of evolved communication networks for its sustainability in the maritime communication environment where various types of ships exist (e.g., traditional ships with legacy radio communication systems such as VHF systems and autonomous surface ships with state-of-the-art radio communication systems such as 5G–Advanced or 6G system). The cyber security is assumed to be guaranteed by the firewall and VPN, etc., for the on-board communication as well as ship-to-shore communication.

This study focuses on the design of an MRGW supporting flexible and scalable interfaces with RAT end-devices using commercial communication networks (e.g., VSAT system or LTE system) and the public Internet.

## 4. Design of an MRGW

### 4.1. Functional Architecture

**Figure 2:**
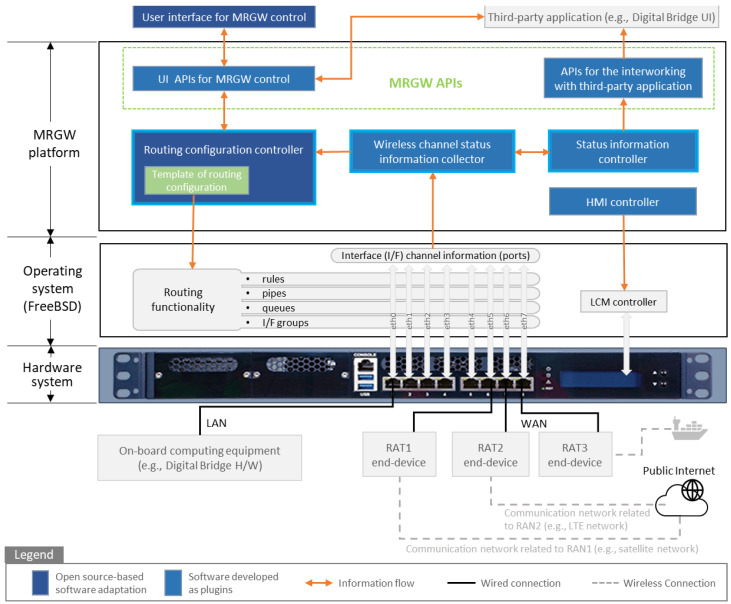
MRGW functional architecture.

An MRGW architecture consisting of a hardware system, an operating system and the MRGW platform is functionally designed, as shown in Figure 2. The open source Unix-like operating system, known as FreeBSD, provides various networking features [46]. For instance, a routing table is dynamically configurable using the MRGW platform through the template of the routing configuration. The MRGW platform implemented on top of the open source software supports the provision of basic networking services and the addition of any user-defined functionality as a plugin.

To support multi-RATs, two functionalities are realized at the MRGW platform. The first functionality is the wireless channel monitoring, developed as a plugin added to the operating system, consisting of following functional components:

- Status information collector of the wireless channel;
- Status information controller;
- Application programming interfaces (APIs) for interworking with third-party applications.

The second functionality is the routing functionality developed to support multi-RATs and the optimal routing selection among accessible RATs, consisting of the following functional components:

- User interface APIs (UI APIs) for MRGW control;
- Routing configuration controller.

In addition, a human machine interface (HMI) controller is developed for the display control in the MRGW hardware system. In addition, APIs for the interworking with third-party applications and UI APIs for MRGW control are developed, through which third-party applications (e.g., the user interface of the digital bridge of the autonomous surface ship) can control the MRGW to provide the wireless channel information of each RAT end-device and select the optimal RAT among the accessible RATs.

#### 4.1.1. MRGW APIs

**Figure 3:**
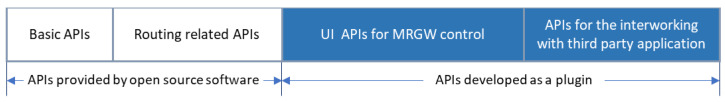
API structure for MRGW access and control.

MRGW APIs provide programmable interfaces for accessing and controlling the MRGW to users and third-party applications. In addition to the various APIs provided by the open source software, the UI APIs for MRGW-control, based on the web interface and interworking APIs for external third-party applications, are provided. The overall API structure for the MRGW access and control is depicted in Figure 3 and is classified into two parts: APIs provided by the open source software and APIs developed as plugins.

#### 4.1.2. Wireless Channel Monitoring Functionality

**Figure 4:**
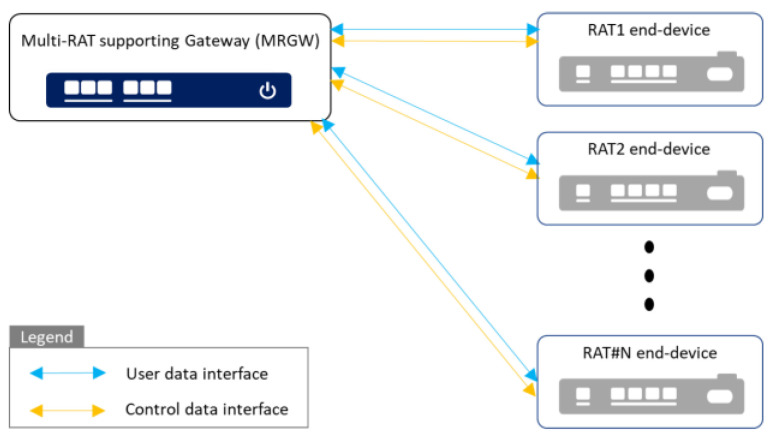
Interfaces between the MRGW and each RAT end-device.

The wireless channel monitoring functionality is realized by two functional components; namely, the wireless channel status information collector and the status information controller in the MRGW platform, as depicted in Figure 2. Accordingly, the wireless channel status information is collected from each RAT end-device and the collected information is provided to other functional components, such as the routing configuration controller or a third-party application (e.g., digital bridge UI), as shown in Figure 2. The interfaces between the MRGW and each RAT end-device are illustrated in Figure 4. The representational state transfer (REST) API and simple network management protocol (SNMP) are used for exchanging the control data between the MRGW and an LTE end-device, as well as between the MRGW and a VSAT end-device, respectively.

#### 4.1.3. Routing Functionality

**Figure 5:**
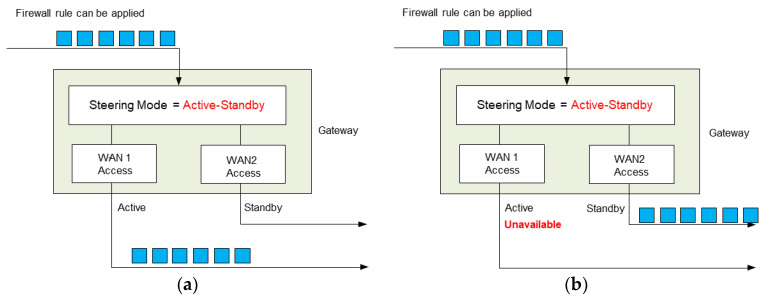
An example of traffic steering operation on Active Standby (**a**) Active access available; (**b**) Active access unavailable.

The MRGW platform adopts routing functionalities supported by the FreeBSD operating system. In addition to those routing functionalities supported by the open source software, the routing configuration controller is implemented as a functional component with several features, such as traffic shaping and traffic steering operations, as shown in Figure 5, to manage user data traffic.

The routing configuration controller receives the wireless channel status information of each RAT and processes this information to select the optimal RAT in the maritime communication environment. The data analysis result provided by the routing configuration controller is delivered to the UI APIs for MRGW control. Furthermore, the basic routing functionality supported by the FreeBSD operating system can be controlled on the MRGW platform by updating the routing configuration information of the FreeBSD operating system through the routing configuration controller’s routing configuration template.

### 4.2. Environment for System Design Verification

**Figure 6:**
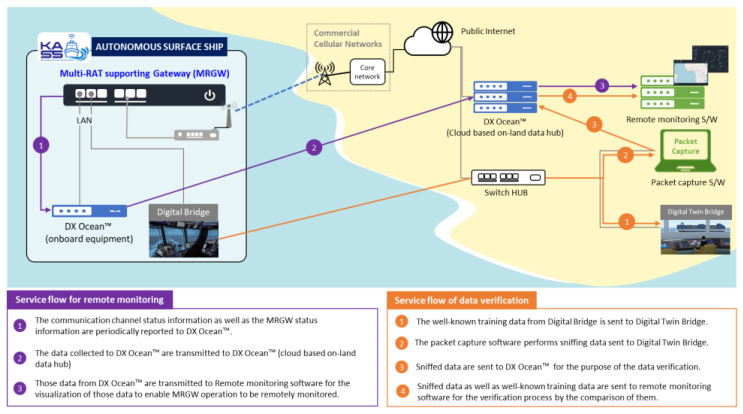
System design for verifying the MRGW’s operation and performance.

The system design for the verification of the MRGW is depicted in Figure 6. The MRGW’s operation and performance are verified using the DX Ocean™, remote monitoring software, and packet capturing software.

To verify the MRGW’s operation and performance, a remote monitoring procedure is implemented using the DX Ocean ™, which enables the information collected from, or created by, the MRGW (e.g., wireless channel status information measured from each RAT end-device or MRGW status information) to be stored in the onboard system as well as a Cloud-based on-land data hub when the communication network is connected between an autonomous surface ship and on-land systems. According to the service flow for remote monitoring, shown in Figure 6, the operation and performance of the MRGW and each RAT end-device can be verified through the visualized data using the remote monitoring software.

To verify the data transmission and reception between the digital bridge of the autonomous surface ship and the digital twin bridge of the on-land control center, the packet capture software sniffs the data exchanged between the digital bridge of the autonomous surface ship and the digital twin bridge of the on-land control center. The packet capture software executes always-on sniffing packets which continuously forward to the DX Ocean™ (Cloud-based on-land data hub). In cases of the stored data being transmitted from the DX Ocean™ (onboard equipment), 30 s is configured as the period of the data transmission from the DX ocean™ (onboard equipment) to the DX ocean™ (Cloud-based on-land data hub). According to the service flow for data verification, shown in Figure 6, whether all of the packets are received is determined by comparing the number of transmitted packets and received (sniffed) packets, and it is also possible to determine whether the MRGW enables the data to be exchanged between the digital bridge and the digital twin bridge using the remote monitoring software, which compares the well-known training data sent from the digital bridge and the sniffed data received by the digital twin bridge for data verification.

## 5. Experimental Validation

### 5.1. Field Experiment Configuration

**Figure 7:**
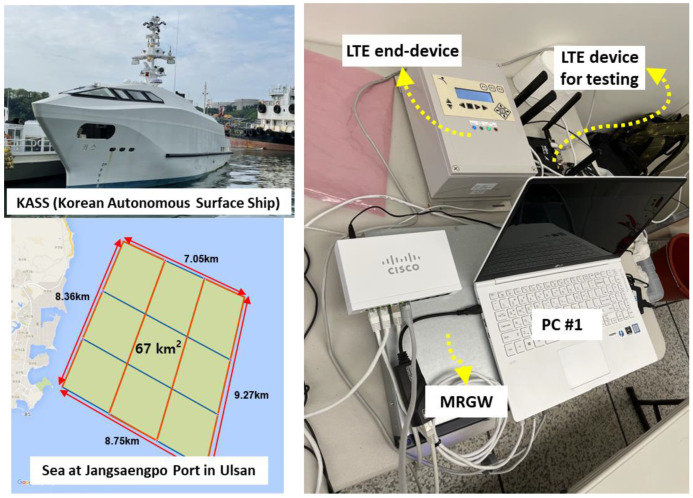
Experimental environment for testing MRGW and LTE end-device in the testbed of KASS at Jangsaengpo port in Ulsan, Republic of Korea.

A field experiment was conducted to verify the operation and performance of the MRGW connected with an LTE end-device and an LTE device for testing in terms of traffic steering between different RATs in the testbed of the autonomous surface ship, known as KASS, plying along the navigation route next to the JangSaengPo port in Ulsan, Korea, as depicted in Figure 7.

**Figure 8:**
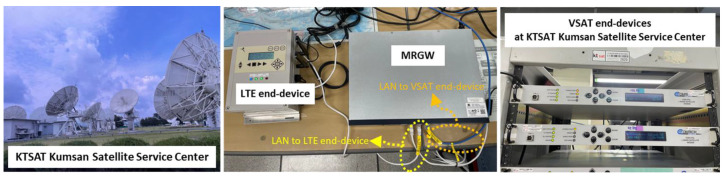
Experimental environment of MRGW interworking between LTE end-device and VSAT end-device at the KTSAT Kumsan satellite service center, in Kumsan, Republic of Korea.

Another field experiment was conducted to verify the operation and performance of the MRGW connected with a VSAT end-device and an LTE end-device in terms of the latency between the different RATs at the KTSAT Kumsan satellite service center in Kumsan, Republic of Korea, as shown in Figure 8 [47]. The VSAT end-device is connected to Republic of Koreasat–6, which is a geostationary satellite operated by KTSAT, with an orbit height of 35,786 km. There is a limitation on the available bandwidth (i.e., 1 Mbps) due to the high cost of the satellite service during the field experiment, so the main purpose of the field experiment is to check the operation of the MRGW and the latency of the satellite communication link.

**Figure 9:**
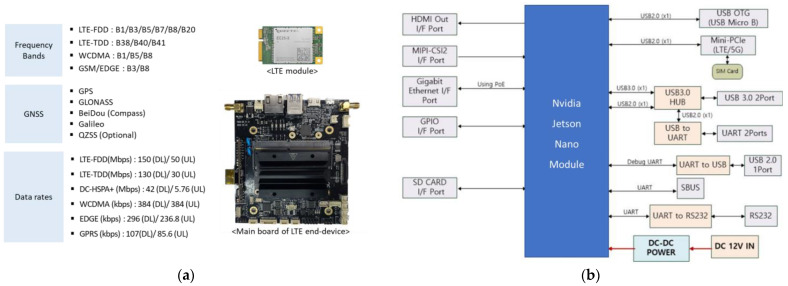
LTE end-device–(**a**) LTE module specification and the main board of LTE end-device; (**b**) Block-diagram of LTE end-device.

For the field experiment, an LTE end-device developed using the LTE and Nvidia Jetson Nano modules is used to enable the MRGW to extract wireless channel status information (e.g., Reference Signal Received Power (RSRP) and Reference Signal Received Quality (RSRQ)) from the LTE end-device. Figure 9 shows the specification of the LTE module and the main board of the LTE end-device.

### 5.2. Test Scenarios and Experiment Outcome Analysis

Three system configurations are considered to test the operation and performance of the MRGW. For the experimental validation, the expected potential operation errors or the breakdown of the MRGW are considered when each experiment execution process for three test scenarios is developed to enable such errors to be identifiable during the experiment by several methods (e.g., testing tools with ping or iperf to identify a hardware malfunction or a configuration error, testing with the DX Ocean™ for the data integrity check). The overall system configuration and experiment outcome analysis for the three test scenarios are provided in following subsections.

#### 5.2.1. Test Scenario 1 for Testing the Switchover Performance of MRGW

Test scenario 1 for testing the switchover performance of the MRGW is to determine whether the MRGW can switch from an active RAT end-device to another standby RAT end-device within 1 s once it detects that the active RAT end-device is disconnected.

**Figure 10:**
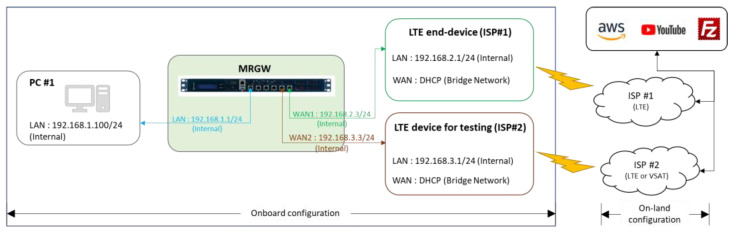
System configuration for the test scenario 1 for evaluating the switchover performance of MRGW.

The overall system configuration for test scenario 1, described in Figure 10, shows the allocated IP addresses used for the validation experiment. The data used for the test scenario are ICMP (Ping) messages generated at PC #1 every second and a public DNS address (i.e., Google public DNS: 8.8.8.8) used as the targeted IP address of the ICMP message.

The switchover performance of the MRGW is verified by performing the following experiment execution processes.

**Figure 11:**
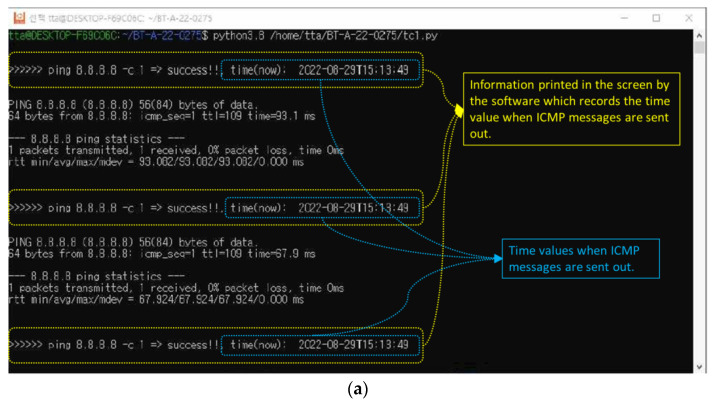

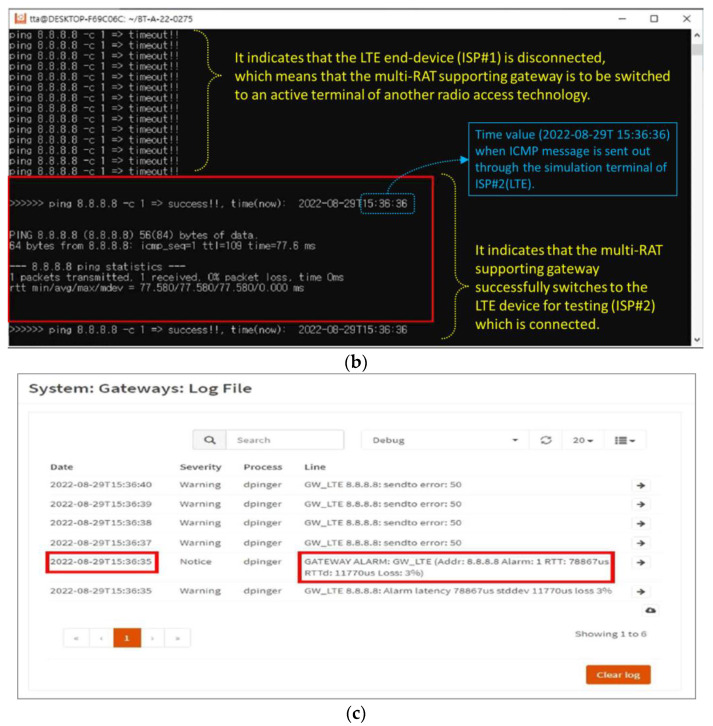
Experiment outcome of test scenario 1–(**a**) Screenshot related to Step 2 of the experimental execution process; (**b**) Screenshot related to Step 4 of the experimental execution process; (**c**) Time value identified from the configuration menu of the MRGW when ‘GATEWAY ALARM occurs because of the disconnection of LTE end-device (ISP#1).

Step 1: The active-standby mode, shown in Figure 5, is configured through the MRGW, and the two RAT end-devices connected to the MRGW are the LTE end-device (ISP#1) and LTE end-device for testing (ISP#2).
Step 2: PC #1 executes the ping program to generate an ICMP message, which is sent to the public DNS address (8.8.8.8), and the information containing the start time of the transmission of the ICMP message is identified on the screen of PC #1 by the software that records the time when the ICMP messages are transmitted (Figure 11a).
Step 3: The power of the LTE end-device (ISP#1) is turned off to create a field experimental environment, where the LTE end-device (ISP#1) is disconnected.
Step 4: The start time of the transmission of the ICMP messages is recorded and identified on the screen of PC #1 once the connection is re-established through the LTE device for testing (ISP#2), instead of the inaccessible LTE end-device (ISP#1) (Figure 11b).
Step 5: The time when the ‘GATEWAY ALARM’ is generated is recorded. This time is identified from the system/gateway/logfile of the user interface for MRGW control, shown in Figure 2 (192.168.1.1) (Figure 11c).

The outcome of the experiment to evaluate the switchover performance of MRGW is presented in Figure 11. The time is printed every time an ICMP message is sent, and Figure 11a shows the examples of the times (blue dotted lines) that were printed every time an ICMP message was sent via the LTE end-device (ISP#1). As soon as the ISP#1 (LTE) end device is disconnected, the MRGW conducts the switchover from the ISP#1 (LTE) end device to the LTE device for testing (ISP#2). Once the MRGW is connected with the LTE device for testing (ISP#2), the time for the successfully sent ICMP message starts to be printed and the first time of an ICMP message sent via the LTE device for testing (ISP#2) is 36 s 36 min 15 h on 29 August 2022, as shown in Figure 11b. The time at which the LTE end-device (ISP#1) is disconnected can be identified only from the user interface for the control of the MRGW by the unit of the second. The time of the disconnection from the LTE end-device (ISP#1) is 35 s 36 min 15 h on 29 August 2022, identified as 2022–08-29T15:36:35, from the user interface for the MRGW control, as shown in Figure 11c. As the MRGW lost the connection from the LTE end-device (ISP#1) at 35 s 36 min 15 h and the MRGW was again connected to the LTE device for testing (ISP#2) at 36 s 36 min 15 h, we verified that the MRGW successfully switched the connection from the LTE end-device (ISP#1) to the LTE device for testing (ISP#2) within 1 s.

#### 5.2.2. Test Scenario 2 for Evaluating the Support for Real-Time Video Streaming Data Provided by MRGW Operation

Test scenario 2 for evaluating the support for the real-time video streaming data provided by the MRGW’s operation is to determine whether the MRGW can establish a connection with an accessible targeted RAT end-device and transmit and receive real-time video streams through the web-RTC server (Janus).

**Figure 12:**
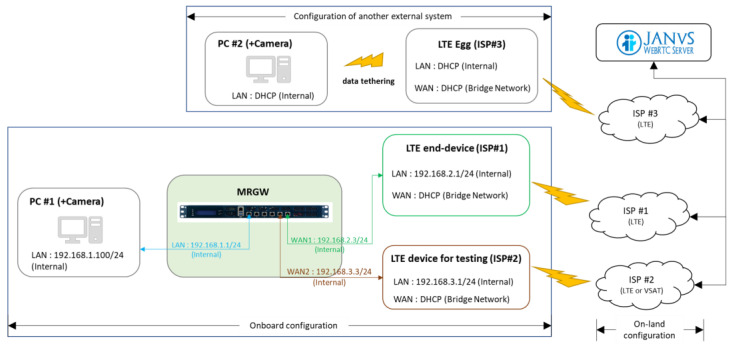
System configuration for test scenario 2 for evaluating the support for real-time video streaming data provided by the MRGW’s operation.

The overall system configuration for test scenario 2, illustrated in Figure 12, shows the allocated IP addresses used for the experimental validation. The Chrome browser is used to access the video room application of the Web-RTC server (Janus) at PC #1 with a camera, and PC #2 with a camera and real-time video streaming data produced from the camera are sent out and received by PC #1 and PC #2, respectively, via the Web-RTC server (Janus).

The MRGW’s operation for supporting real-time video streaming data is verified by performing the following experiment execution processes.

Step 1: The active-standby mode, shown in Figure 5, is configured using the MRGW, and the two RAT end-devices connected to the MRGW are the LTE end-device (ISP#1) and the LTE device for testing (ISP#2) for the interworking test.
Step 2: PC #1 accesses the Web-RTC server (Janus) through the Chrome browser and activates the camera and microphone to produce real-time video streaming.
Step 3: PC #2 accesses the Web-RTC server (Janus) through the Chrome browser and activates the camera and microphone to produce real-time video streaming.
Step 4: PC #1 and PC #2 determine whether the real-time video streaming produced from the activated camera and microphone is displayed through the video room of the Web-RTC server (Janus) with no service interruption.

**Figure 13:**
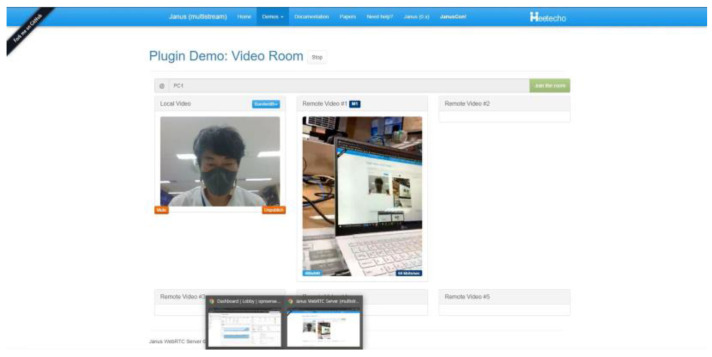
Experiment outcome of test scenario 2 (Chrome browser screenshot of the video room through Web-RTC server (Janus)).

The outcome of the experiment for evaluating the MRGW’s operation for supporting real-time video streaming data is depicted in Figure 13, which includes two video streaming images of the video room application of the Web-RTC server (Janus). The left image is captured from the camera of PC #1 and the right one is captured from the camera of PC #2. They are displayed from the video room of the Web-RTC server (Janus) at PC #1 because the MRGW successfully exchanges the video streaming data via the LTE end-device (ISP#1). Based on the experiment outcome, we verified that the MRGW successfully ensured that the real-time video streaming was provided through the Web-RTC server (Janus).

#### 5.2.3. Test Scenario 3 for Evaluating the Operation of MRGW in Interworking with the DX Ocean™

Test scenario 3 for evaluating the operation of the MRGW in interworking with the DX Ocean™ is to determine whether the MRGW interworks with the DX Ocean™; the information collected by the DX Ocean™ (onboard equipment) deployed in the KASS is sent out and delivered to the DX Ocean™ (Cloud-based on-land data hub) through the MRGW and each RAT end-device.

**Figure 14:**
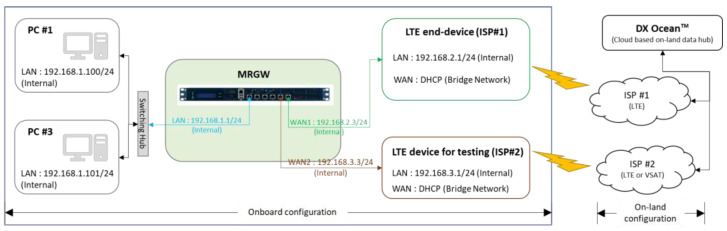
System configuration for test scenario 3 for evaluating the operation of MRGW in interworking with DX Ocean™.

The overall system configuration for test scenario 3, illustrated in Figure 14, shows the allocated IP addresses used for the experimental validation. The API related to the wireless channel status information collector, shown in Figure 2, is assumed to be identified and the DX Ocean™ software is installed and executed at PC #1.

The operation of the MRGW in interworking with the DX Ocean™ is verified by performing the following experiment execution processes.

Step 1: The active-standby mode, shown in Figure 5, is configured using the MRGW, and the two RAT end-devices connected to the MRGW are the LTE end-device (ISP#1) and the LTE device for testing (ISP#2) for the interworking test.
Step 2: The information related to the configuration of the MRGW is identified from the menu of the user interface for MRGW control, depicted in Figure 2.
Step 3: The DX Ocean™ software at PC #1 stores the information collected from the API related to the wireless channel status information collector, shown in Figure 2, and the information regarding a ship’s location obtained from the positioning system deployed in the ship.
Step 4: The information stored in the DX Ocean™ (onboard equipment) is transmitted to the DX Ocean™ (cloud-based on-land data hub) through the MRGW via either the LTE end-device (ISP#1), if accessible, or the LTE device for testing (ISP#2), if accessible.

**Figure 15:**
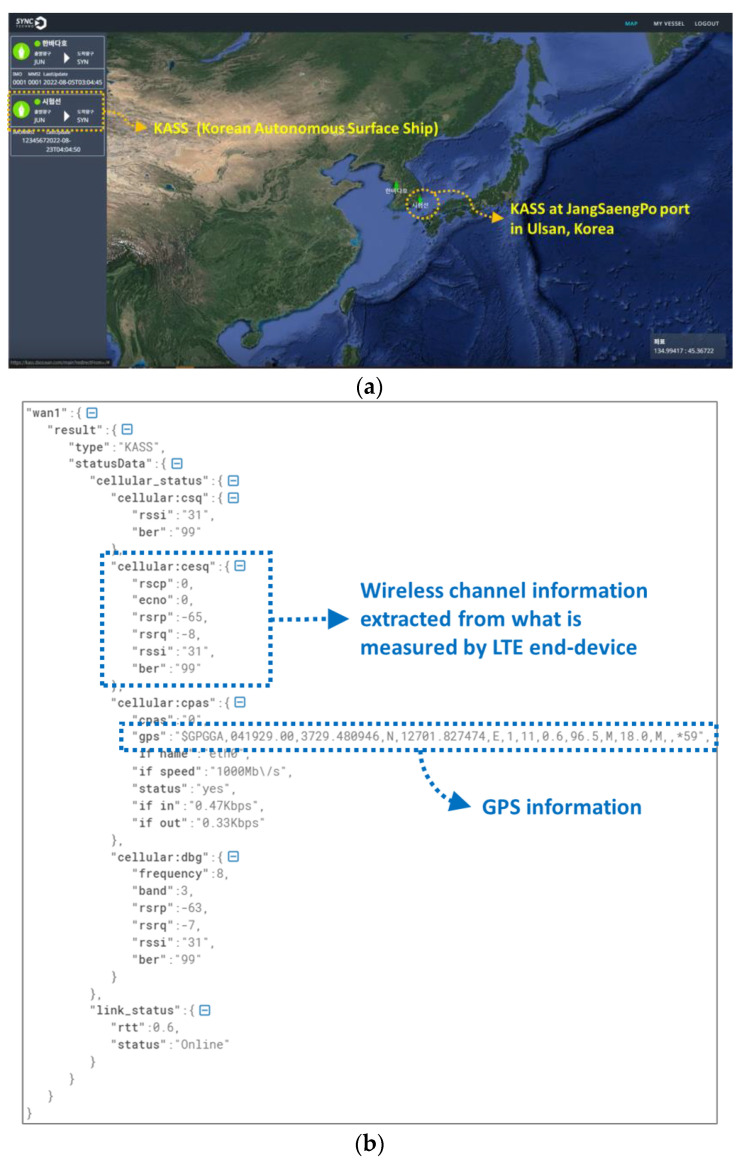
Experiment outcome of test scenario 3 (**a**) DX Ocean™ software screenshot (**b**) Sample of ISP#1(LTE) wireless channel status information collected from the MRGW and stored at DX Ocean™ (onboard equipment and cloud-based on-land data hub).

The experiment outcome for test scenario 3 is shown in Figure 15. As shown in Figure 15a, the DX ocean™ shows the ship location based on the GPS information which the MRGW obtains from the LTE end-device (ISP#1) and subsequently sends it to the DX Ocean™ (onboard equipment). The DX Ocean™ (onboard equipment) sends the stored data out to the DX Ocean™ (cloud-based on-land data hub) every 30 s. The period of the data sent out from the DX Ocean™ (onboard equipment) to the DX Ocean™ (cloud-based on-land data hub) is configurable. Figure 15b is the sample of collected data related to the wireless channel status information from the MRGW. Based on the experiment outcome, we found that the information collected from the MRGW is successfully stored in the DX Ocean™ (onboard equipment and cloud-based on-land data hub).

### 5.3. Experimental Outcome of Wireless Performance

**Figure 16:**
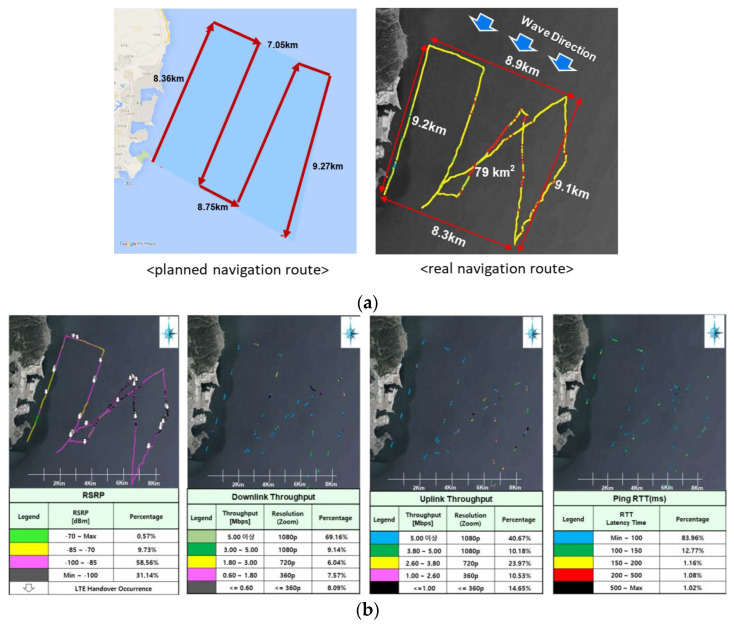
Experiment outcome of LTE end-device (**a**) navigation route in front of JangSaengPo port in Ulsan, Republic of Korea; (**b**) measured LTE performance.

The wireless performance of the LTE end-device is shown in Figure 16. The real navigation route was revised compared to the planned navigation route because of the wave direction due to the worse weather condition at sea for the reduction in the safety risk during the testing. In terms of the RSRP, 68.86% of the total navigation route shows values ≥ –100 dBm. In terms of the downlink throughput, 92% of the total navigation route shows values ≥ 0.6 Mbps. In terms of the uplink throughput, 85% of the total navigation route shows values ≥ 1 Mbps. In terms of the latency, 98% of the total navigation route shows values ≤ 200 ms.

The wireless performance of the LTE end-device was inferior compared to that of the wireless performance of the LTE end-devices in urban areas. However, the performance may have been hampered by the operator policy in terms of the difference in network operation between urban and maritime/rural areas.

**Figure 17:**
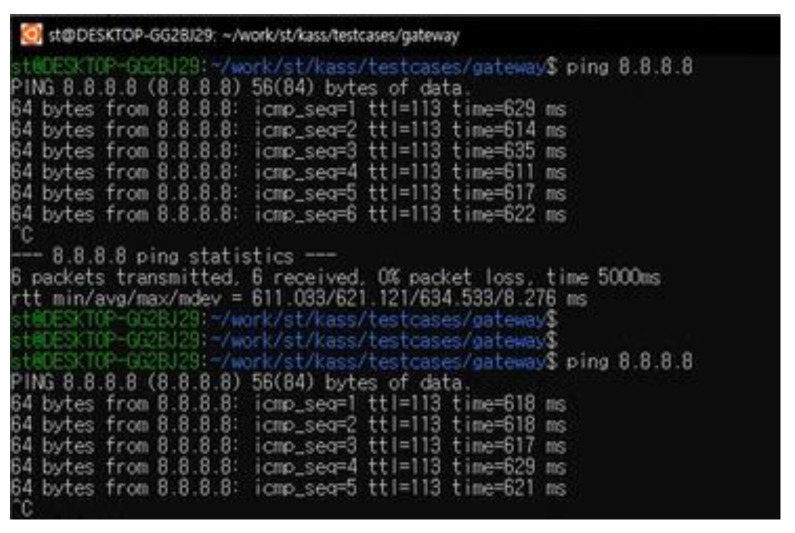
Round trip time measured from VSAT end-device at KTSAT Kumsan satellite service center, in Kumsan, Republic of Korea.

The round trip time measured from the VSAT end-device is shown in Figure 17. The worst case round trip time propagation delay of a GEO satellite with transparent payload is 541.46 ms [48], but the measured round trip time of the satellite was more than 610 ms during the testing at the KTSAT Kumsan satellite service center in Kumsan, Republic of Korea.

**Figure 18:**
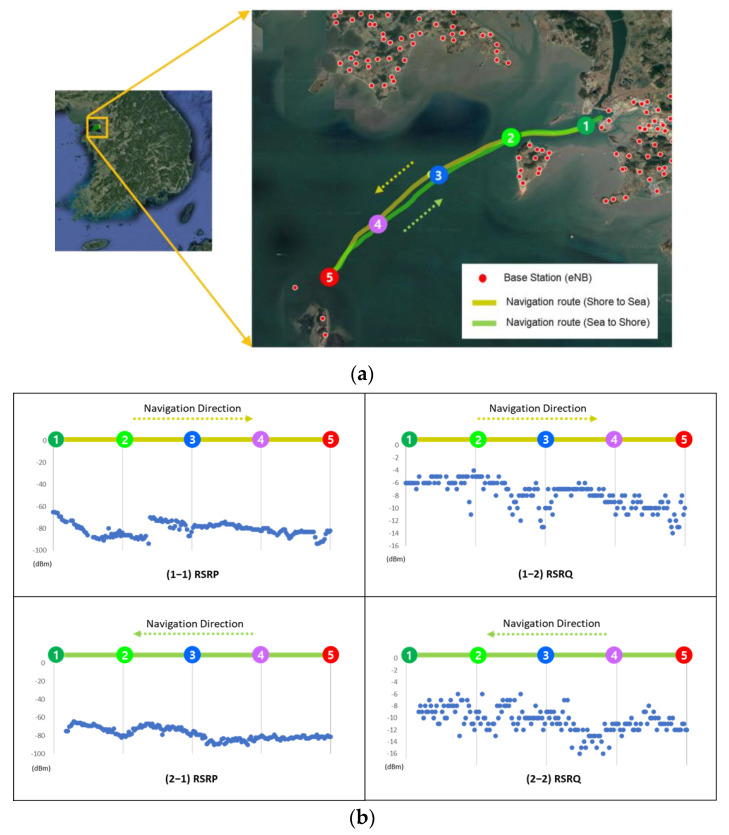
Experimental outcome of LTE end-device (**a**) navigation route in front Jeongok Port in Hwasung city, Republic of Korea; (**b**) measured LTE performance (RSRP, RSRQ).

An additional experiment was performed using the fishing boat in front of the Jeongok Port at the west sea of Republic of Korea and the MRGW collected the wireless channel status information (i.e., RSRP, RSRQ, GPS information) measured from the LTE end-device. The wireless performance measured from the LTE end-device is shown in Figure 18. As shown in Figure 18a, the navigation route is identically selected for the shore-to-sea direction and the sea-to-shore direction to have the effect of the repeated experiments. The weather condition was good, and it was a sunny day, which means that waves did not cause any impact to degrade the wireless performance at sea. As shown in Figure 18b, two of the graphs (i.e., (1–1) and (2–1)) have a similar RSRP pattern and another two graphs (i.e., (1–2) and (2–2)) also have a similar RSRQ pattern. Based on the experiment outcome, we can assume that little change occurs to the wireless channel status in cases of a sunny day, which means that our experimental validation may be meaningful despite the limited number of the experiments.

## 6. Conclusions

Herein, we proposed a design for a novel MRGW and conducted corresponding experiments to verify the operation and performance of the MRGW based on three test scenarios with different system configurations. The experiment outcomes indicate that the MRGW has the capability of providing the seamless connection for an autonomous surface ship through multiple RAT end-devices in the maritime communication environment. Based on the MRGW’s capability, proven by the experimental validation in the real maritime communication environment, it is important to continue to enhance the MRGW to support new upcoming end-devices (e.g., 5G end-device supporting both cellular access and satellite access) based on more evolved RATs, such as 5G-Advanced and 6G. Our research is expected to be continuously carried out during the second step of the KASS project [49], from the year 2023 to the year 2025, to enhance the functions of the MRGW and address any abnormal potential errors by applying assurance framework, such as a FMECA (Failure modes, effects, and criticality analysis) mechanism, and performing more experimental validations for three years in the testbed of autonomous surface ships in Ulsan, Republic of Korea. IMT-based technologies continue to be evolved to support the requirements of the vertical industries, including the maritime sector. The service requirements related to the digital transformation of the maritime sector, including the autonomous surface ship, are important to be provided during the global standardization of 5G-Advanced and beyond in 3GPP. Subsequent future works, as well as the research outcome of this paper, are expected to be leveraged to identify the gap analysis of existing commercial communication technologies and develop maritime requirements that should be satisfied by 5G or 6G systems that are possible to be employed as the fundamental communication platform for the digitalization and mobilization of the maritime sector.

In the near future, an end-device with both satellite and cellular access (e.g., 5G or LTE) is expected to be launched in markets as a 5G satellite was first specified to be integrated into 5G systems in 3GPP specifications in March 2022. However, further research is needed to enhance the capabilities of the current 5G satellite, particularly in terms of support for low earth orbit satellites, which are always in motion, not stationary. New opportunities and challenges for improving the MRGW can be found in research areas related to the support of multiple radio accesses, including low earth orbit satellites integrated into the 5G system.

The digital bridge system controlling the navigation of an autonomous surface ship and its counterpart on-land control center are to be deployed and operated 24/7/365 in the near future and the quantity of data is expected to increase rapidly. The data generated for the experimental verification are not the same as the data expected to be generated during the real operation of the digital bridge of the autonomous surface ship and the on-land control center. Additional research on the enhancement of the MRGW needs to be conducted to address how to flexibly manage the uplink and downlink bandwidth because the bandwidth allocated for the uplink transmission is generally lower than that of the downlink transmission in commercial cellular networks.

## Data Availability

Not applicable.
